# Prediction learning in adults with autism and its molecular correlates

**DOI:** 10.1186/s13229-021-00470-6

**Published:** 2021-10-06

**Authors:** Laurie-Anne Sapey-Triomphe, Joke Temmerman, Nicolaas A. J. Puts, Johan Wagemans

**Affiliations:** 1 Department of Brain and Cognition, Leuven Brain Institute, KU Leuven, 3000 Leuven, Belgium; 2 Leuven Autism Research (LAuRes), KU Leuven, 3000 Leuven, Belgium; 3 Department of Forensic and Neurodevelopmental Sciences and the Institute of Psychiatry, Psychology, and Neuroscience, Sackler Institute for Translational Neurodevelopment, King’s College London, London, SE5 8AF UK; 4 MRC Centre for Neurodevelopmental Disorders, King’s College London, London, SE5 8AF UK

**Keywords:** ASD, GABA, Glutamate, Glutathione, Magnetic resonance spectroscopy, Prediction, Prior, Probabilistic learning, Uncertainty, Volatility

## Abstract

**Background:**

According to Bayesian hypotheses, individuals with Autism Spectrum Disorder (ASD) have difficulties making accurate predictions about their environment. In particular, the mechanisms by which they assign precision to predictions or sensory inputs would be suboptimal in ASD. These mechanisms are thought to be mostly mediated by glutamate and GABA. Here, we aimed to shed light on prediction learning in ASD and on its neurobiological correlates.

**Methods:**

Twenty-six neurotypical and 26 autistic adults participated in an associative learning task where they had to learn a probabilistic association between a tone and the rotation direction of two dots, in a volatile context. They also took part in magnetic resonance spectroscopy (MRS) measurements to quantify Glx (glutamate and glutamine), GABA + and glutathione in a low-level perceptual region (occipital cortex) and in a higher-level region involved in prediction learning (inferior frontal gyrus).

**Results:**

Neurotypical and autistic adults had their percepts biased by their expectations, and this bias was smaller for individuals with a more atypical sensory sensitivity. Both groups were able to learn the association and to update their beliefs after a change in contingency. Interestingly, the percentage of correct predictions was correlated with the Glx/GABA + ratio in the occipital cortex (positive correlation) and in the right inferior frontal gyrus (negative correlation). In this region, MRS results also showed an increased concentration of Glx in the ASD group compared to the neurotypical group.

**Limitations:**

We used a quite restrictive approach to select the MR spectra showing a good fit, which led to the exclusion of some MRS datasets and therefore to the reduction of the sample size for certain metabolites/regions.

**Conclusions:**

Autistic adults appeared to have intact abilities to make predictions in this task, in contrast with the Bayesian hypotheses of ASD. Yet, higher ratios of Glx/GABA + in a frontal region were associated with decreased predictive abilities, and ASD individuals tended to have more Glx in this region. This neurobiological difference might contribute to suboptimal predictive mechanisms in ASD in certain contexts.

**Supplementary Information:**

The online version contains supplementary material available at 10.1186/s13229-021-00470-6.

## Background

Humans are sensitive to the contingent relationships between cues and outcomes in social or non-social contexts, such as the chances of having someone interact with you after making eye contact or seeing rain soon after hearing thunder. These associations rely on mechanisms of inference [1, 2], whereby the brain tries to predict the most likely outcome by computing probabilities based on past experiences. Cast in the Bayesian framework, making optimal inferences requires adjusting our prior knowledge when there is a relevant discrepancy (i.e. prediction error) between our prediction and the actual outcome [1]. The weight of prediction errors depends on the relative precisions of top-down predictions and bottom-up sensory inputs. The precision of prediction errors should be high when mismatches represent informative changes, but low when signalling noise. Minimizing prediction errors, and therefore surprise, can be achieved through optimal predictive mechanisms [3] that track different forms of uncertainty. Not only does the expected uncertainty (e.g. probabilistic cue-outcome association) need to be tracked, but also the unexpected uncertainty (e.g. unsignalled reversal of the associations) [4].

In daily life, dealing with uncertainty can be challenging, especially for individuals with Autism Spectrum Disorders (ASD) who report a high intolerance of uncertainty [5–7]. Indeed, in addition to the two core symptoms of ASD that are impairments in social communication and interaction, and restricted and repetitive behaviours [8], autistic individuals often report difficulties in tolerating unexpected changes. More broadly, autistic individuals could perceive the world as being more unpredictable than neurotypicals (NT) [9]. Accounts of ASD formulated from a Bayesian perspective suggest an atypical functioning of the predictive brain in ASD [10–15]. Their increased inflexibility or their needs for a routine could be a way to restore some predictability (i.e. to compensate for feelings of constant unpredictability). More precisely, the first Bayesian accounts of ASD suggested that perception might not be biased by priors so much, either because prior precision is low [10] or because sensory precision is high [11]. Another hypothesis suggested a high and inflexible precision of prediction errors in ASD (HIPPEA, 12). If prediction errors are not flexibly modulated and are always given a high weight in ASD, it would lead to suboptimal prior updating and to (almost) constant sensations of surprise.

These hypotheses of ASD were elaborated after re-interpreting the existing literature on learning and perception in ASD within the Bayesian framework, but did not come from tasks that were specifically designed to test these hypotheses. Since the formulations of these hypotheses, several studies attempted to test them more directly. A recent systematic review evidenced some differences between ASD and NT individuals in predictive learning and predictive responses [16]. They found that most of the studies investigating the predictability of repeated stimuli showed reduced habitation in ASD (e.g. 17, 18). Most studies on structural priors (i.e. priors learned over long time scales) reported no differences between NT and ASD (e.g. 19, 20), whereas studies on contextual priors (i.e. priors learned over shorter time scales) gave more heterogeneous results in ASD. For instance, low-level perceptual tasks in autistic adults showed slower prior learning in ASD [21] or more inflexible priors [22]. Results of associative learning tasks in ASD often depend on context. In uncertain contexts, the ability to learn and update associations was typical in autistic children (colour-reward association task, 23), but atypical in autistic adults (tone-visual outcome association, 24). In their study [24], a tone (high or low) was probabilistically associated with an outcome (face or a house) and participants simply had to report what the outcome was. By modelling the response times (modulated by the expectedness of the outcome), the authors showed that autistic adults tended to overestimate the volatility of the environment [24]. Yet, a recent study suggested that this might be restricted to subgroups of autistic individuals [25]. In another associative learning study where adults had to learn an association between a tone and a rotation direction in a very uncertain context, autistic adults managed to learn a prior, but failed to update it after an unexpected change in contingency [26]. Another study relying on a large cohort of children and adults performing a probabilistic reversal learning task also showed poorer performance in ASD and reduced flexible behaviours [27]. Finally, another probabilistic reward learning task in a volatile environment showed that having more autistic traits was associated with worse performance [28]. Overall, these behavioural studies tend to indicate that priors can be learned in ASD, but maybe with a different dynamic, more inflexibility or with decreased abilities in people with ASD or high autistic traits.

While the investigation of prediction learning in ASD has made good progress at the behavioural level (for a review: 15), little is known about its underlying neurobiological mechanisms in ASD. As mentioned above, ASD might be characterized by an atypical precision of priors [10], sensory inputs [11] or prediction errors (i.e. sensory/prior balance, 12). Precision is thought to be encoded by the post-synaptic gain of superficial neurons signalling prediction errors [29, 30]. This gain quantifies the effect of a presynaptic input on a post-synaptic output, and mostly relies on glutamatergic NMDA receptors [29, 30]. According to this framework, an atypical encoding of precision in ASD should be associated with an abnormal (glutamatergic) neuromodulation. Top-down predictions would be mediated by slow NMDA glutamatergic receptors [31], in particular in the prefrontal cortex [32]. Bottom-up prediction errors would be mediated by fast AMPA glutamatergic receptors and GABA_A_ receptors [31]. Another hypothesis suggests that rapid glutamatergic and GABAergic neurotransmission would represent prediction errors, whereas slower neuromodulators (such as acetylcholine) would encode the precision of prediction errors [33, 34]. All these hypotheses point towards the key role of glutamate (excitatory) and GABA (inhibitory) in predictive mechanisms. Abnormal concentrations or signalling of these neurotransmitters would alter the ability to encode the precision of predictions or prediction errors, and so, to optimally learn predictions. Consistently, computational simulations suggested that an increased glutamate/GABA ratio would be consistent with a decreased influence of contextual priors [35]. Given the role of glutamate and GABA in predictive mechanisms and the hypotheses of altered predictive abilities in ASD, the question arises whether abnormal glutamate/GABA signalling could be related to the Bayesian accounts of ASD.

Interestingly, abnormalities in the glutamatergic and GABAergic neurotransmission were found in ASD, including atypical neurotransmitter concentration, receptor density or enzyme dysregulation (e.g. [36, 37]). On the one hand, some studies support the *hyperglutamate theory* in ASD and are based on increased glutamate concentrations in the serum and plasma, on the upregulation of NMDA receptors in animal models induced with valproic acid, or on the behavioural ameliorations triggered by antagonists of glutamatergic receptors [38]. On the other hand, some other studies support the *hypoglutamate theory* in ASD and rely on dysfunction of certain glutamatergic receptors and on beneficial effects of glutamatergic agonists in ASD [38]. Studies using the in vivo and non-invasive method of magnetic resonance spectroscopy (MRS) in autistic adults showed that the level of Glx (glutamate and glutamine) depended on the brain region: Glx was either increased (amygdala–hippocampal complex [39], auditory cortex [40], sensorimotor cortex [41], anterior cingulate cortex (ACC) [42], cerebellum [43]), decreased (ACC [44, 45], central nuclei [46, 47]) or not different from NT (frontal regions [46, 48–51], parietal regions [39, 44, 46], occipital regions [49, 52], cingulate cortex [50, 53], thalamus [44]). Regarding GABA, most of the evidence is in favour of a decreased action of GABA in ASD [37, 54]. In autistic adults, MRS studies mostly found decreased GABA levels (sensorimotor cortex [55], supplementary motor area [56], ACC [43], cerebellum [43]) or no group differences (frontal regions [47, 49, 51, 52, 56, 57], occipital regions [49, 52, 55, 56], auditory cortex [52, 58], ACC [59], striatum [47]).

It is also important to note that glutamate is converted into GABA by the glutamic acid decarboxylase (GAD, either the 65 or 67 isoforms), whose concentration was found to be reduced in ASD [60]. This would lead to an increase in glutamate and decrease in GABA in ASD, which is consistent with the results reported above. Consistently, high Glx/GABA ratios measured with MRS were associated with more autistic traits (e.g. superior temporal cortex [61, 62]). Glutamate is also a precursor of glutathione (GSH), which can be simultaneously measured with edited MRS [63]. GSH is a key component regulating oxidative stress and several other cellular and genetic pathways that contribute to protect cells. GSH therefore plays a neuroprotective role in the brain, and could, for instance, be released to protect neurons when there is an excess of glutamate [64]. An imbalance in the GSH redox system could contribute to the neuropathology of ASD (e.g. [64]).

To sum up, several findings indicate an imbalance in glutamate and/or GABA in autistic adults. As these neurotransmitters are hypothesized to play a key role in encoding predictions and prediction errors, an alteration of the glutamate/GABA signalling could underlie the difficulties in making predictions, as observed at the behavioural level in ASD. Glutamate is a precursor of GABA but also of GSH, a neuroprotective molecule. Although there is no specific hypothesis directly relating GSH to prediction learning, it contributes to the same metabolic cycle as glutamate and GABA. An imbalance in GSH could alter neuronal functions and indicate abnormal Glutamate metabolism. Our exploratory approach is therefore to determine the existence of a deficit/excess in GSH, which could contribute to shedding light on the functioning of brain regions hypothesized to be involved in encoding priors.

In order to better understand prediction learning in autistic adults and to investigate its neural correlates, we conducted a behavioural study combined with MRS measurements of Glx, GABA and GSH. NT and autistic adults performed an associative learning task where a high or low pitch tone was predictive of a clockwise or counterclockwise rotation of a pair of dots (same paradigm as in [66]). There was some expected uncertainty about the cue-outcome association (i.e. 75% of the trials presenting the main association), as well as a part of unexpected uncertainty as the association could suddenly reverse. Participants had to make explicit predictions about the outcome and to report what they perceived. In a subset of ambiguous trials, the dots did not rotate, but we expected participants to be biased by their predictions (i.e. to report the expected rotation). Therefore, there was an explicit measure of prediction learning, as well as a measure of prior bias. Participants also filled in questionnaires assessing their autistic traits or symptoms and their intolerance of uncertainty. Finally, Glx, GABA and GSH were simultaneously quantified using edited MRS in a low-level perceptual region (i.e. the medial occipital cortex) and in a higher-level region hypothesized to play a role in learning predictions [67, 68], (i.e. the right inferior frontal gyrus, IFG).

The current study had several objectives. First, we aimed at better characterizing prediction learning in ASD and relating it to the autistic symptomatology. For this purpose, we assessed whether autistic adults managed to learn and update their predictions and whether they were biased by their expectations. The Bayesian hypotheses suggesting hypo-prior [10] or high sensory precision [11] in ASD would predict a decreased prior bias and a decreased performance to make predictions. However, in light of the recent empirical findings cited above, we predicted that autistic individuals would learn a prior but may be more inflexible to update it. Within the Bayesian literature of ASD, defaults in making accurate predictions are supposed to underlie the autistic symptomatology and to account for their higher intolerance of uncertainty. Therefore, we predicted that the percentage of correct predictions would be negatively correlated with the questionnaire scores assessing social difficulties, atypical sensory sensitivity and intolerance of uncertainty. We also measured whether this predictive ability varied along the autism spectrum using the Autism-spectrum Quotient, as autistic traits are continuously distributed in the population [69] and were correlated with performance in a probabilistic learning task [28]. Second, our other main goal was to investigate the neuromolecular correlates of prediction learning in ASD using MRS. Precisely, we expected the Glx/GABA ratio to be correlated with the ability to make predictions in lower- and higher-level regions. We were especially interested in assessing whether there would be such a correlation in the IFG, as it would be involved in encoding predictions and prediction errors [67, 68], and that atypicalities were reported in this region in ASD. To better characterize the functioning of these two regions in ASD, we also compared the concentrations in Glx, GABA and GSH between groups. Given the MRS literature, we hypothesized that these concentrations would not differ in ASD versus NT in the occipital cortex. There are no published MRS results in the right IFG in autistic adults; yet, given its potential role in making predictions and the altered abilities observed in ASD, we hypothesized that Glx levels might be increased and/or GABA levels decreased, which may be associated with an increase in GSH.

## Methods

### Participants

Participants were 26 neurotypical (NT) adults and 26 adults with a diagnosis of ASD. The demographic characteristics of the two groups are shown in Table 1. The two groups were matched in age, sex ratio, handedness ratio and total intellectual quotient. Inclusion criteria were being between 18 and 50 years old, reporting normal or corrected-to-normal hearing and vision. Exclusion criteria were having contra-indication for MRI, having a total intellectual quotient below 70 at the Wechsler Adult Intelligence Scale IV (Wechsler, 2008), or scoring above 32 at the Autism-Spectrum Quotient (AQ, Baron-Cohen et al., 2001) for NT participants (to reduce the chances of recruiting someone who may have ASD without being diagnosed). Autistic participants received their diagnoses from a multidisciplinary Expertise Centre for Autism (University Hospitals of KU Leuven) in a standardized way according to the criteria of the Diagnostic and Statistical Manual of mental disorders 5 (DSM-5, American Psychiatric Association, 2013). Autistic participants were recruited via this expertise centre and via the LAuRes (Leuven Autism Research) consortium website if they had signed up to be volunteers for upcoming studies. NT participants were recruited via the University of Leuven or via acquaintances. None of the NT participants reported having a comorbidity or being under medication. Five autistic participants reported having comorbidities (ADHD [4], dyslexia [2], Gilles de la Tourette [1]) and eleven reported taking one or several medication (Abilify (2), Asaflow (1), Bufonix (1), Celecoxib (1), Cipralexa (1), Deanxit (1), Depakine (1), Escitalopram (1), Fluoxetine (1), Fluoxone (1), Hydrea (1), L-Thyroxine (3), Medikinet (1), Melatonine (1), Montelucast (1), Notrilen (1), Redomex (1), Ritalin (1), Trazadone (1), Venlafaxine (1), Welbutrin (1)). Note that none of the medications directly targeted the GABAergic or glutamatergic pathways, except for Depakine.

**Table 1:** Demographic characteristics and questionnaire scores of the participants

	NT group	ASD group	*p*
Number of participants	26	26	–
Male/female number	13/13	13/13	*ns*
Age (years)	30.9 (± 8.3)	32.2 (± 9.5)	*ns*
Left/right-handed	2/24	5/21	*ns*
Total IQ score	113.9 (± 12.3)	112.1 (± 16.5)	*ns*
AQ score	13.0 (± 6.2)	32.1 (± 8.7)	***
SRS score	33.4 (± 13.3)	84.0 (± 24.5)	***
IU score	27.9 (± 8.4)	42.7 (± 6.6)	***
GSQ score	34.5 (± 16.7)	53.3 (± 21.2)	***

The table presents the group means (± standard deviations). IQ: Intelligence Quotient (Wechsler Adult Intelligence Scale IV); AQ: Autism-spectrum Quotient; SRS: Social Responsiveness; IU: intolerance of uncertainty; GSQ: Glasgow Sensory Questionnaire. Note that IQ data from four NT participants are missing as they were not native Dutch speakers. Regarding the AQ, all the NT participants scored below the 32 cut-off threshold defined in [66], and below the 26 cut-off threshold suggested in [97] except for one NT participant who had an AQ of 29. ns: non-significant (*p* > .05), ***p < .001

This study was approved by the medical Research Ethical Committee UZ/KU Leuven. Participants provided written informed consent before the start of the experiment.

### Overall procedure

Prior to the experiment measuring prediction learning, participants filled in online questionnaires: the Autism-spectrum Quotient (AQ) [71], the Social Responsiveness Scale 2nd edition (SRS-2) [72], the Glasgow Sensory Questionnaire (GSQ) [73] and a short version of the Intolerance of Uncertainty scale (IU) [74] (see the scores in Table 1). The goal was to assess if difficulties in making predictions were related to autistic traits (AQ), social difficulties (SRS), atypical sensory sensitivity (GSQ) and intolerance of uncertainty (IU).

Participants performed a short training of the main task (7 trials) and then were installed in the MR scanner. A T1-weighted anatomical scan and two MR spectroscopy acquisitions were obtained, followed by the main task (functional MRI data were acquired as part of another study). After leaving the MR scanner, participants performed a confidence rating task (48 trials) and completed a short debriefing questionnaire (Additional file 1). MRI and MRS data were acquired on a 32 head coil 3 T Philips Achieva system at the University Hospital of Leuven.

### Experimental paradigm

#### Main task

##### Trial structure

**Fig. 1:**
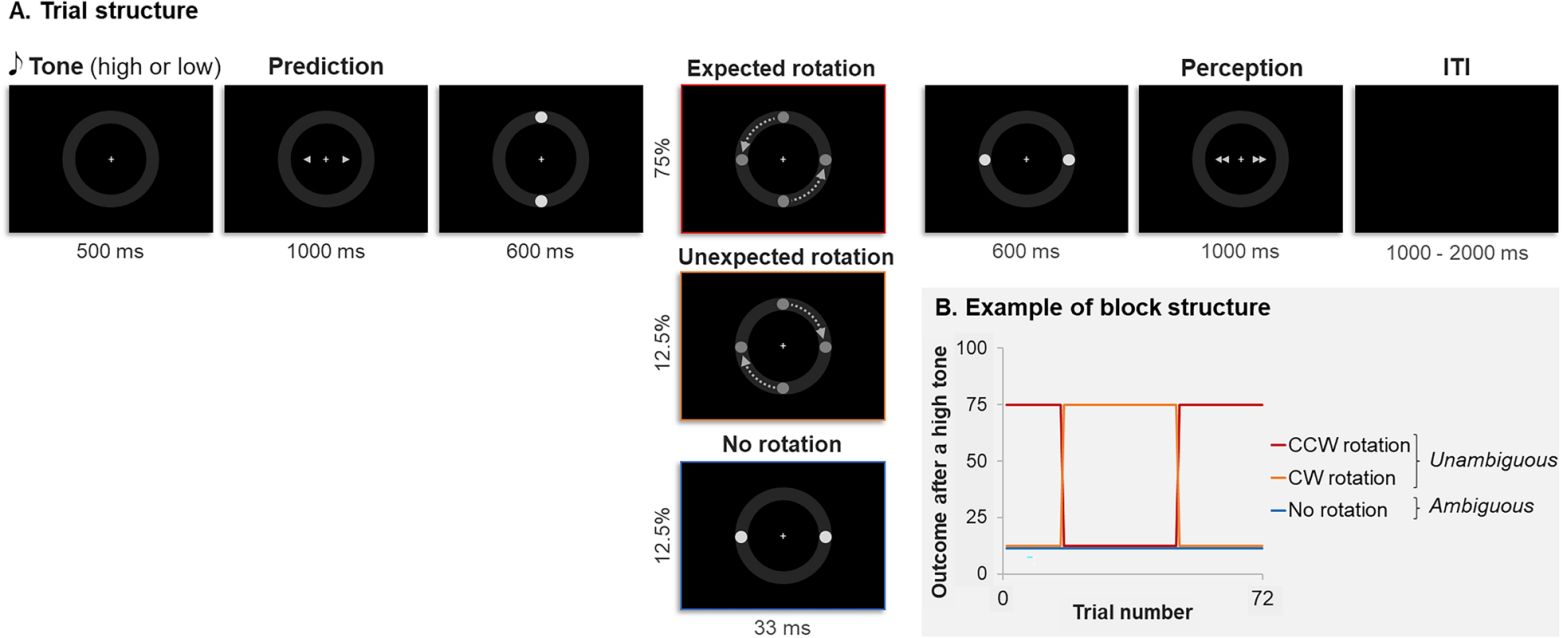
Experimental paradigm. **a** Trial structure. After hearing a low or a high pitch tone, participants had to predict the rotation direction of a pair of dots, and then to report their percept. The two dots could either rotate in the expected or unexpected direction (unambiguous trials) or simply appear in their vertical and then horizontal position without rotating (ambiguous trials). **b** Example of a block structure. There was a probabilistic association between the tone and the rotation direction, which reversed every 16, 24 or 32 trials. For instance, the plot shows the probability of having a certain outcome (CCW or CW rotation, or no rotation) given that a high tone that was heard. *CW* clockwise, *CCW* counterclockwise, *ITI* inter-trial interval

In the associative learning task (Fig. 1a), participants first heard a high (576 Hz) or low (352 Hz) tone for 500 ms. After a jitter of 100 to 300 ms, they saw the *prediction* response screen for 1000 ms, displaying a right and left arrow. Participants were asked to click on the right or left button of the MRI response box, if they thought that the tone was predictive of a clockwise (CW—right) or counterclockwise (CCW—left) dot rotation, respectively. The chosen arrow turned red. Then, two dots appeared at their vertical position for 600 ms, made a CW or CCW rotation within 33 ms and remained at their horizontal position for 600 ms. The *perception* response screen appeared for 1000 ms and displayed a right and left double-arrow. Participants had to report whether they perceived a CW or CCW rotation using the right or left button, respectively, and the chosen double-arrow turned red. The inter-trial interval lasted for 1000 to 2000 ms (uniform distribution). This paradigm was based on a study by Weilnhammer and colleagues [66].

In *unambiguous trials*, the pair of dots rotated, whereas in *ambiguous* trials the two dots did not rotate but simply appeared in their vertical and then horizontal positions.

Participants were given the following instructions: “At the beginning of each trial, you will hear a high or a low tone. This tone can predict the direction in which two dots will turn (CW or CCW). The association between the sound and the rotation direction can change during this experiment”.

##### Block structure

Participants completed five blocks of 72 trials (total of 360 trials). Half of the trials started with a high pitch tone and the other half with a low pitch tone. Each run consisted of 9 ambiguous trials (12.5% of the trials) and 63 unambiguous trials (87.5% of the trials). There were 75% of expected unambiguous trials (i.e. presenting the main tone-rotation association) and 12.5% of unexpected unambiguous trials (i.e. presenting the least frequent association). Among the unambiguous trials, 50% were showing a CW rotation and 50% a CCW rotation. The order of the trials was pseudo-randomized so that these percentages (75%, 12.5%, 12.5%) remained the same across eight successive trials. Each block consisted of a 16 trial-long, 24 trial-long and 32 trial-long subblock, whose order was randomized. The main contingency remained the same within a subblock but reversed when changing of subblock (i.e. every 16, 24 or 32 trials, see Fig. 1b).

#### Confidence rating task

As in Weilnhammer et al. [66], the main task was followed by a confidence task to ensure the perceptual quality of the ambiguous trials (vs. unambiguous trials). The structure of this task was the same as the main task, but participants also gave a confidence response. The confidence rating screen showing the options “1. Very sure”, “2. Quite sure”, “3. Quite unsure”, “4. Very unsure” was displayed for 2600 ms after the perception screen. Participants used the numbers 1 to 4 on the keyboard to report how confident they were about their perception response. There were 48 trials, divided into two subblocks of 24 trials, with 50% of ambiguous trials and 50% of unambiguous trials.

## MRI and MRS acquisitions

### Anatomical scan

A high-resolution T1-weighted anatomical scan was acquired with a MPRAGE sequence (200 contiguous coronal slices, voxel size = 1 × 1 × 1 mm^3^, TR = 9.7 ms, TE = 4.6 ms, field of view = 256 × 240 × 200 mm^3^, acquisition matrix = 256 × 238, acquisition time = 4 min 35 s). This structural image was used to position the MRS voxels.

### Magnetic resonance spectroscopy

**Fig. 2:**
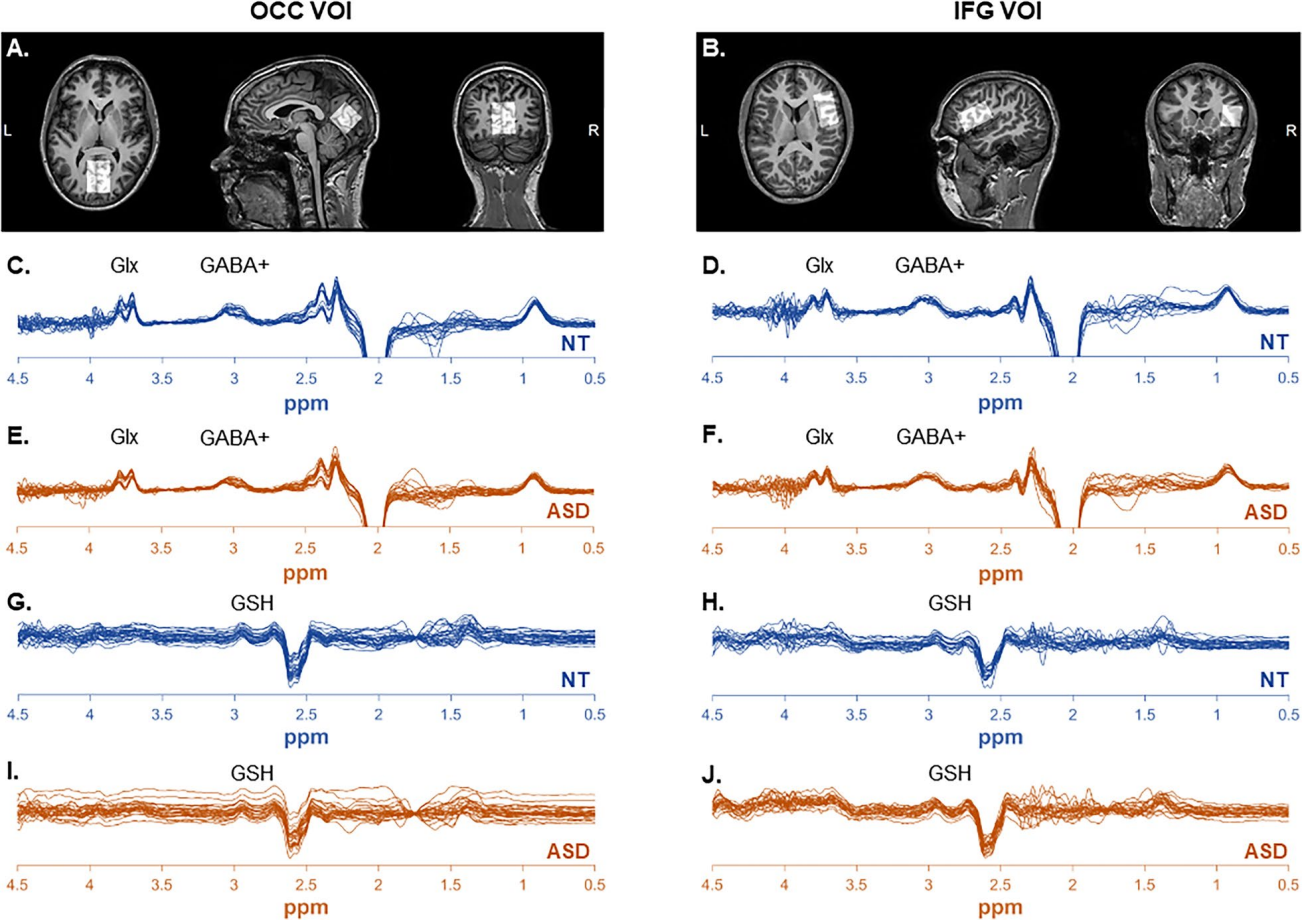
Glx, GABA+ and GSH spectra in the occipital VOI (OCC, left column) and right inferior frontal gyrus (IFG, right column). **a, b** Examples of MRS voxel localization centred over the medial occipital cortex (**a**) and right IFG (b). **c–f** Glx and GABA + spectra in the OCC VOI (**c** NT, **e** ASD) and IFG VOI (**d** NT, **e** ASD). **g–j** GSH spectra in the OCC VOI (**g**: NT, **i**: ASD) and IFG VOI (**h**: NT, **j**: ASD). Blue: spectra in NT participants. Orange: spectra in ASD participants

Single voxel MR spectra were acquired from two volumes of interest (VOI): one located in the occipital cortex (OCC) and one located in the right inferior frontal gyrus (IFG). The OCC VOI was chosen as a low-level perception region (receiving the visual input), while the IFG VOI was chosen as a higher-level region, as it may play a key role in predictive processes [e.g. 67, 68]. The OCC VOI was acquired first and the IFG VOI second. The OCC VOI (3 × 3 × 3 cm^3^) was placed in the medial of the coronal slice, dorsal to the cerebellum (Fig. 2a). The IFG OCC (4 × 2.5 × 2.5 cm^3^) was centred on the triangularis part of the right IFG, and the longer dimension of the VOI was dorsal to the temporal cortex (Fig. 2b). Note that the IFG VOI slightly overlapped with the ventral parts of the middle frontal and precentral gyri.

MR spectra were acquired using the Hadamard Encoding and Reconstruction of MEGA-Edited Spectroscopy (HERMES) sequence [63, 75, 76], allowing for simultaneous quantification of GABA + , Glx (glutamine and glutamate) and GSH (glutathione). The MRS acquisition parameters were set as follows: 320 averages, TR = 2000 ms, TE = 80 ms, 2048 data points, 2000 Hz spectral width, MOIST water suppression, 90°excitation/180°refocusing pulses, 20 ms editing pulses at frequencies of 4.56 ppm for GSH and 1.9 ppm for GABA. Interleaved Water Reference correction was used to limit the effect of scanner drift [77]. Twenty unsuppressed water reference scans (at TE = 80 ms) were also acquired. The MRS acquisition lasted for 10 min 56 s per VOI. Data were exported as.sdat/.spar.

## MRS data analysis

HERMES MRS spectra were analysed using Gannet 3.1 [78], implemented in MATLAB 2020b. Frequency domain spectra were frequency- and phase-corrected using spectral registration (specRegHERMES [79]). Preprocessing of the MRS data also included a 3 Hz line broadening filter (exponential apodization function), fast Fourier transformation, time averaging, frequency and phase corrections based upon fitting of the Choline and Creatine signals, and pairwise rejection of data for which fitting parameters were greater than three standard deviations from the mean. GABA and Glx were fitted with a three Gaussian model using nonlinear least square fitting between 2.79 and 4.10 ppm. The unsuppressed water spectrum was modelled between 3.8 and 5.6 ppm using a single Gauss-Lorentzian mode with phase and linear baseline parameters, also using nonlinear least-squares fitting. The GSH spectrum was estimated from 2.25 and 3.5 ppm using a single Gaussian to model the 2.95 ppm GSH signal with four Gaussians to model co-edited unwanted signals and a nonlinear baseline.

Upon a careful visual inspection of all the MR spectra, and the criterium that the fit error for Glx and GABA + should not exceed 15%, some participants were excluded from the analyses (see Table 2). The mean fit error, the full-width at half-maximum (FWHM), the water drift and signal-to-noise ratios (SNR) of the remaining participants are given in Table 2. None of these quality metrics differed between groups except for the GABA + concentration in the OCC VOI and the GSH concentration in the IFG VOI. The tissue fractions did not differ between groups in any VOI (Table 2). Individual spectra are shown in Fig. 2c–j.

**Table 2:** MRS quality metrics, tissue fractions and concentrations

	Occipital VOI (OCC)			Inferior frontal gyrus (IFG)		
	NT	ASD	*p*	NT	ASD	*p*
*Number of MRS datasets*						
GABA+	24	25	*na*	22	24	*na*
Glx	20	22	*na*	15	16	*na*
GSH	26	26	*na*	20	23	*na*
*Fit error (%)*						
GABA+	6.4 (± 2.8)	8.2 (± 2.8)	*	7.5 (± 2.0)	6.8 (± 1.5)	*ns*
Glx	3.9 (± 0.8)	4.4 (± 1.1)	*ns*	5.3 (± 1.3)	5.0 (± 1.1)	*ns*
GSH	7.3 (± 2.0)	8.3 (± 2.6)	*ns*	14.5 (± 6.3)	10.9 (± 4.2)	*
*Signal to noise ratio*						
GABA+	13.0 (± 3.8)	11.5 (± 3.3)	*ns*	12.9 (± 2.3)	11.9 (± 2.1)	*ns*
Glx	19.9 (± 4.5)	21.2 (± 6.1)	*ns*	17.6 (± 4.7)	16.6 (± 4.0)	*ns*
GSH	10.1 (± 1.9)	9.8 (± 1.8)	*ns*	9.0 (± 1.8)	8.6 (± 1.3)	*ns*
Water drift	0.03 (± 0.01)	0.03 (± 0.01)	*ns*	0.02 (± 0.00)	0.02 (± 0.00)	*ns*
*Full-width at half-maximum*						
GABA+	21.9 (± 2.7)	22.5 (± 2.5)	*ns*	20.7 (± 2.1)	21.0 (± 2.2)	*ns*
Glx	14.2 (± 2.6)	13.6 (± 2.0)	*ns*	15.8 (± 3.8)	17.2 (± 5.2)	*ns*
GSH	8.7 (± 1.0)	8.9 (± 0.9)	*ns*	10.5 (± 2.7)	11.1 (± 2.5)	*ns*
*Tissue fractions*						
Grey matter	65% (± 4)	66% (± 3)	*ns*	54% (± 5)	54% (± 4)	*ns*
White matter	25% (± 3)	24% (± 3)	*ns*	38% (± 6)	38% (± 5)	*ns*
Cerebrospinal fluid	10% (± 2)	10% (± 3)	*ns*	8% (± 3)	8% (± 3)	*ns*
*Concentrations*						
GABA + (i.u.)	2.94 (± 0.72)	2.75 (± 0.61)	*ns*	2.85 (± 0.53)	2.83 (± 0.61)	*ns*
Glx (i.u.)	9.08 (± 2.90)	9.35 (± 2.60)	*ns*	8.12 (± 1.43)	9.90 (± 3.14)	*
GSH (i.u.)	0.93 (± 0.17)	0.92 (± 0.23)	*ns*	1.13 (± 0.40)	1.16 (± 0.36)	*ns*
Glx/GABA + ratio	3.75 (± 2.44)	3.73 (± 1.52)	*ns*	2.91 (± 0.68)	3.59 (± 1.50)	*ns*

The table presents the group means (± standard deviations). Note that after exclusion of some participants leading to the numbers reported in the table, none of the demographic characteristics differed between these NT and ASD subgroups. na: not applicable, ns: not significant (*p* > .05), *p < .05. *p*: *p* values of the Student's *t* tests comparing the two groups

The VOIs were co-registered to the anatomical T1-weighted image and segmented in Gannet through SPM12 in order to obtain the fractions of grey matter (*f*_*GM*_), white matter (*f*_*WM*_) and cerebrospinal fluid (*f*_*CSF*_). GABA, Glx and GSH concentrations were quantified relative to the unsuppressed water signal, and corrected for tissue fractions using *α* correction (e.g. [GABA]_αcor_ = $\frac{{\left[ {GABA} \right]}}{{f_{GM} + \alpha f_{WM} }}$, with *α* = 0.5 as per [80] which assumes a GM/WM ratio for GABA of 2:1 based on prior literature [81]). Additional parameters included an assumed visible water concentration of 50,000 mM, an editing efficiency of 0.5 and T1- and T2-specific values for both GABA and water as described in [80] where T1 and T2 values were estimated to be different between GM and WM. Gannet does not incorporate tissue compartment-specific values for GABA as these are currently not available, but for water tissue-specific values were used for GM, WM, and CSF, with different MR-visible concentrations as reported [82]. Note that macromolecules at 3 ppm are co-edited with GABA, so GABA levels are reported as GABA+ [83] and that an additional MM factor of 0.5 was used for the quantification of the estimated concentrations, as recommended by [84]. The same approach was used for GSH due to lack of published information for GSH [85]. Metabolite concentrations are given in institutional units [i.u.]. The tissue fractions are reported in Table 2.

## Statistical analyses

Note that in the Results section, all results are presented as mean (± standard deviation). The threshold for statistical significance was set at *p* < 0.05. All Student's t tests were two-tailed. Effect sizes are reported as Cohen’s *d*, with the following interpretations regarding effect sizes: *d* = 0.01: very small, *d* = 0.20: small, *d* = 0.50: medium, *d* = 0.80: large, *d* > 1.20: very large [86, 87]. A Pearson’s *r* of 0.10 is considered as a small effect, 0.30 as a medium effect and 0.50 as a large effect. When multiple comparisons were performed, we also indicated whether the correlations remained significant after False Discovery Rate (FDR) correction. Statistical analyses were performed using R (version 4.0.3, http://www.r-project.org/).

### Behavioural data

Demographic data were compared between groups using Student's t tests and χ^2^ tests. Percentages of correct predictions and of ambiguous trials perceived according to the current contingency were compared to chance level using one-sample t tests with *μ* = 0.50. Between-group comparisons were performed using two-sample t tests. Accuracy and response times were analysed using repeated-measure ANOVAs, with group as a between-subject factor (NT vs. ASD) and condition as a within-subject factor (ambiguous vs. unambiguous trials, or expected vs. unexpected trials). In the ANOVAs, the independent variables were the groups and the conditions, while the dependent variables were the percentages of correct predictions, the percentages of ambiguous trials perceived according to the current contingency, and the response times. ANOVAs were performed using the function aov (R, http://www.r-project.org/) which fits an analysis of variance model. When significant effects were found, paired or two-sample Student's t tests were used as post-hoc tests. Median response times were calculated at the individual level, and the mean of these median response times across participants is reported. In the confidence task, a mean confidence rating was calculated: the 1 to 4 scale (*Very sure* to *Very unsure* scale) was transformed into a 100% to 0% certainty scale. Correlations were assessed using Pearson’s correlation tests.

### MRS data

The variables of interest in the MRS analyses were the Glx, GABA + and GSH concentrations, as well as the Glx/GABA + ratio. In addition, we extracted several MRS quality metrics (reported in Table 2) to ensure the quality of the data and that they did not differ between groups. The Glx, GABA + and GSH concentrations were compared between groups using Student's t tests. The Glx/GABA + was correlated with the mean percentage of correct predictions using Pearson correlation tests. Correlations between metabolite concentrations and questionnaire scores were also assessed using Pearson correlation tests.

## Results

### Behavioural results

#### Prediction response in the main task

**Fig. 3:**
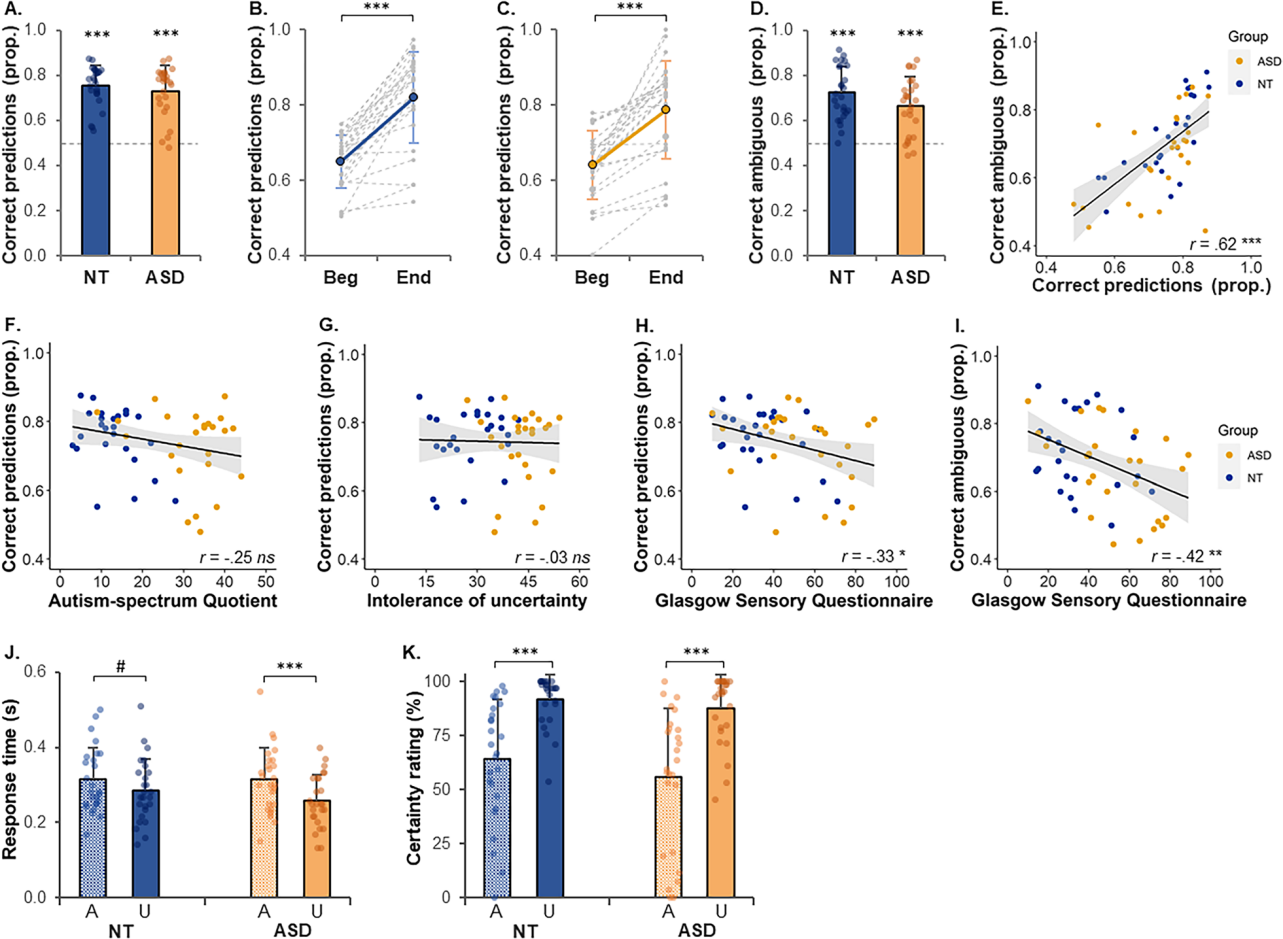
Behavioural results of the main task.** a** Proportion of correct predictions. The dotted line indicates chance level. **b, c** Proportion of correct predictions at the beginning (first 8 trials) and end (last 8 trials) of a subblock in the NT group (**b**) and ASD group (**c**). Grey dotted lines show individual responses; thick lines indicate the group mean. **d** Proportion of ambiguous trials perceived according to the current contingency. The dotted line indicates chance level. **e** Correlation between the proportions of correct predictions and of ambiguous trials perceived according to the current contingency in the NT (blue) and ASD (orange) groups. **f–h** Correlations between the proportion of correct predictions and questionnaire scores from the AQ, IU and GSQ. **i** Correlation between the proportion of ambiguous trials perceived according to the current contingency and the GSQ. **j** Response time (mean at the group level, median at the individual level) to give a perception response for ambiguous (A) and unambiguous (U) trials. **k** Mean certainty rating for ambiguous (A) and unambiguous (U) trials). In all the plots, error bars indicate standard deviations. Blue: NT data, Orange: ASD data. Prop.: Proportion. ^#^*p* = .06, *p < .05, **p < .01, ***p < .001

Both groups succeeded at predicting the dot pair rotation, as the mean percentage of correct predictions was 76% (± 9) in the NT group and 73% (± 11) in the ASD group (Fig. 3a), and were different from chance level (NT: *t*(25) = 14.6, *p* < 0.0001, *d* = 2.86; ASD: *t*(25) = 10.7, *p* < 0.0001, *d* = 2.10). There were no group differences in accuracy (*p* = 0.38).

In order to assess whether both groups updated their predictions, we conducted a repeated-measure ANOVA with the between-subject factor *group* (NT vs. ASD) and the within-subject factor *part* (beginning of a subblock = 8 first trials after a contingency change, vs. end of a subblock = 8 last trials of a subblock) on the percentage of correct predictions as a dependent variable. There was a main effect of *part* (*F*(1,50) = 130.4, *p* < 0.0001), no effect of group effect nor interaction effect (p-values > 0.40). The percentage of correct predictions increased from 65% (± 7) to 82% (± 12) in NT (*t*(25) = 8.8, *p* < 0.0001, *d* = 1.72, Fig. 3b) and from 64% (± 9) to 79% (± 13) in ASD (*t*(25) = 7.4, *p* < 0.0001, *d* = 1.45, Fig. 3c).

#### Perception response in the main task

##### Perception response in unambiguous trials

Both groups got 99% (± 1) of correct responses in unambiguous trials (no group difference, *p* = 0.57). We conducted a repeated-measure ANOVA on the percentages of correct perception responses (dependent variable), with *group* as a between-subject factor and *expectedness* (expected vs. unexpected rotation) as a within-subject factor. There was an effect of *expectedness* (*F*(1,50) = 9.3, *p* < 0.01), with a slightly higher accuracy in expected (99% ± 1) than unexpected (98% ± 3) trials (*t*(51) = 3.1, *p* < 0.01, *d* = 0.43). There was no group effect nor interaction effect (p-values > 0.80).

##### Perception response in ambiguous trials

The percentages of ambiguous trials perceived as following the current contingency (Fig. 3d) were above chance level in NT (72% ± 12, *t*(25) = 9.7, *p* < 0.0001, *d* = 1.90) and ASD (66% ± 13, *t*(25) = 6.6, *p* < 0.0001, *d* = 1.29), suggesting that NT and ASD participants were biased by their expectations. There was no significant group difference (*t*(50) = 1.7, *p* = 0.095, *d* = 0.47). The percentages of ambiguous trials perceived according to the current contingency and of correct predictions were positively correlated (Fig. 3e) (entire sample: *r* = 0.62, *p* < 0.0001).

A repeated-measure ANOVA assessed the effect of *group* as a between-subject factor and *part* (beginning vs. end) as a within-subject factor on the percentage of ambiguous trials perceived according to the contingency (dependent variable). There was a *part* effect (*F*(1,50) = 8.2, *p* < 0.01), but no group nor interaction effects (p-values > 0.20). The percentage of ambiguous trials perceived according to the current contingency increased from 64% (± 16) to 73% (± 19) (*t*(51) = 2.9, *p* < 0.01, *d* = 0.40). Note that within group, this increase was significant in NT (from 65% ± 12 to 76% ± 19, *t*(25) = 2.9, *p* < 0.01, *d* = 0.58), but not in ASD (from 62% ± 19 to 70% ± 18, *t*(25) = 1.4, *p* = 0.17, *d* = 0.28).

##### Unambiguous versus ambiguous trials

Response times were longer in ambiguous than unambiguous trials (Fig. 3j) in the ASD group (314 ms ± 86 vs. 258 ms ± 70, *t*(25) = 4.8, *p* < 0.0001, *d* = 0.93), and did not differ significantly in NT (314 ms ± 86 vs. 286 ms ± 84, *t*(25) = 2.0, *p* = 0.060, *d* = 0.39).

#### Correlations between behavioural data and questionnaires

To investigate whether the symptoms of ASD were associated with difficulties to make predictions, we investigated correlations between the percentage of correct predictions and autistic traits (AQ), social difficulties (SRS), atypical sensory sensitivity (GSQ) and intolerance of uncertainty (IU). The percentage of correct predictions was not significantly correlated with the AQ (*r* = − 0.25, *p* = 0.069, Fig. 3f), SRS (*r* = − 0.26, *p* = 0.067) and IU (*r* = − 0.03, *p* = 0.85, Fig. 3g), and was significantly correlated with the GSQ (*r* = − 0.33, *p* = 0.018, Fig. 3h). These correlations were performed on the entire sample (across both groups).

The percentage of ambiguous trials perceived according to the main contingency was significantly correlated with the GSQ (entire sample: *r* = − 0.42, *p* = 0.002, Fig. 3i), so that individuals with a more atypical sensory sensitivity were less biased by their expectations.

After adjusting for multiple comparisons using FDR correction, the two correlations with the GSQ remained significant.

#### Confidence rating task

A repeated-measure ANOVA assessing the effects of *ambiguity* (ambiguous vs. unambiguous trials) as a within-subject factor and *group* as a between-subject factor on certainty ratings revealed an effect of ambiguity (*F*(1,50) = 40.0, *p* < 0.0001), but no group effect nor interactions (p values > 0.17). Ambiguous trials were rated as more uncertain than unambiguous trials (60% ± 30 vs. 90% ± 14, *t*(51) = 6.4, *p* < 0.0001, *d* = 0.88, Fig. 3k).

### Correlations between prediction learning abilities and Glx/GABA + ratios

As the glutamate/GABA balance is hypothesized to be involved in signalling predictions and prediction errors, we explored whether the Glx/GABA + ratio was correlated with the ability to learn predictions and whether correlations were mostly driven by Glx and/or GABA.

**Fig. 4:**
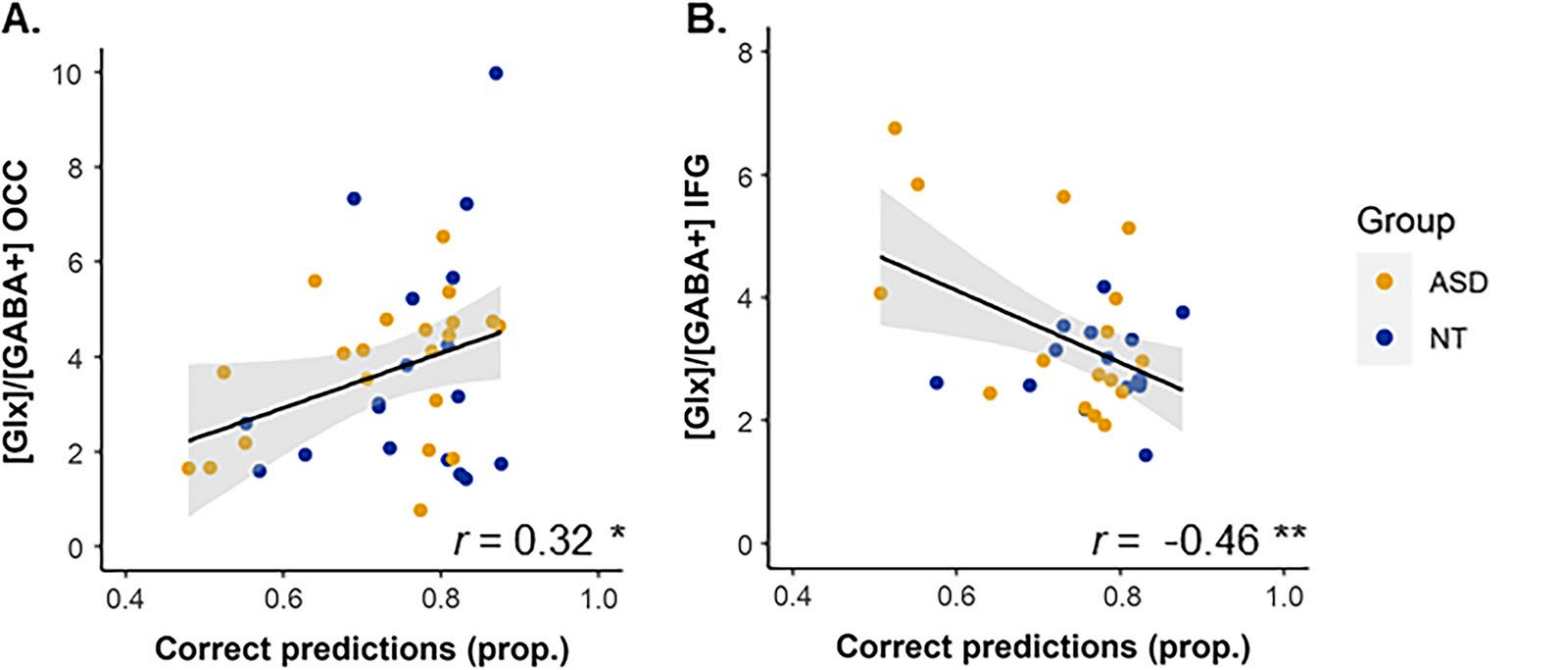
Correlations between proportions of correct predictions and Glx/GABA + ratios in the occipital (OCC) (**a**) and right inferior frontal gyrus (IFG) (**b**) VOIs. Prop.: proportion. *p < .05, **p < .01.

In the OCC VOI, the percentage of correct predictions was positively correlated with the Glx/GABA + ratio (*r* = 0.32, *p* = 0.048, Fig. 4a). Within group, this correlation did not reach the significance level in ASD (*r* = 0.40, *p* = 0.070) or NT (*r* = 0.29, *p* = 0.25). This correlation appeared to be driven both by Glx (entire sample: *r* = 0.30, *p* = 0.051) and GABA + (entire sample: *r* = − 0.27, *p* = 0.064).

In the IFG VOI, the percentage of correct predictions was negatively correlated with the Glx/GABA + ratio (entire sample, *r* = − 0.46, *p* = 0.010, Fig. 4b). Within group, this correlation was significant in ASD (*r* = − 0.55, *p* = 0.028), but not in NT (*r* = 0.04, *p* = 0.89). This correlation seemed more driven by Glx (entire sample: *r* = − 0.38, *p* = 0.034) than by GABA + (entire sample: *r* = 0.16, *p* = 0.29).

These two correlations with the Glx/GABA + ratio remained significant after correction for multiple comparisons using FDR corrections (adjusted p-values: *p* = 0.048 in OCC, *p* = 0.020 in IFG).

Note that if the 11 ASD participants taking medication are removed from the analyses, the correlation coefficients remain close to the ones reported above (OCC VOI: *r* = 0.31, *p* = 0.088, IFG VOI: *r* = − 0.40, *p* = 0.049).

### Glx and GABA concentrations

The mean metabolite concentrations are reported in Table 2.

**Fig. 5:**
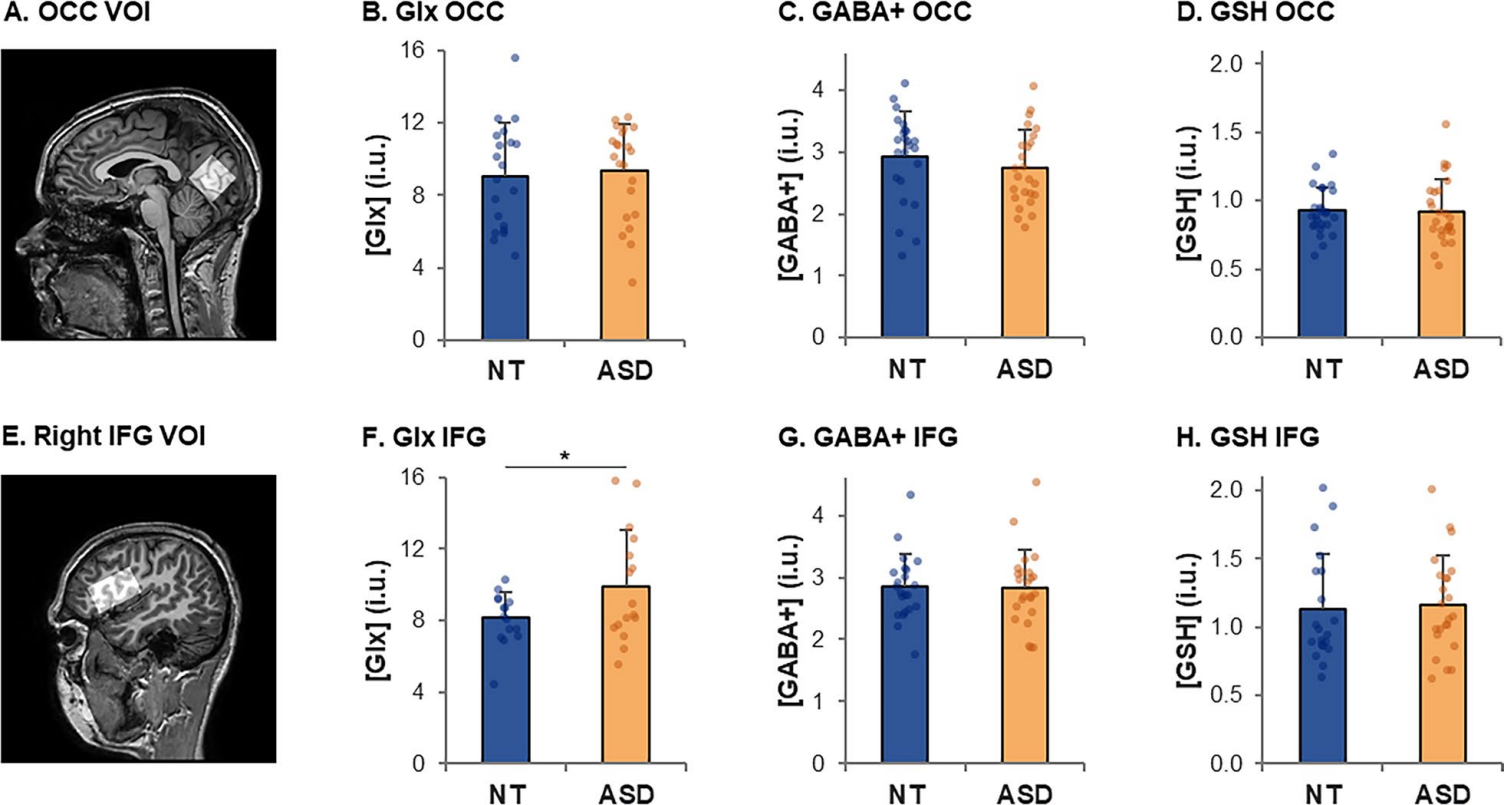
Estimated metabolite concentrations in the occipital VOI (first raw) and right inferior frontal gyrus (IFG, second raw). Metabolite concentrations are given in institutional units (i.u.) and were corrected for tissue fractions. Error bars indicate standard deviations. *p = .05

In the OCC VOI, the Glx and GABA concentrations did not differ between groups (Glx: *p* = 0.75, Fig. 5b, GABA + : *p* = 0.31, Fig. 5c), and neither did the Glx/GABA + ratio (*p* = 0.98).

In the IFG VOI, the Glx concentrations were higher in the ASD group than in the NT group (ASD: 9.90 ± 3.14 i.u. vs. NT: 8.12 ± 1.43 i.u.; *t*(21) = 2.0, *p* = 0.05, *d* = 0.72, Fig. 5f). Note that this result is just at the significance level but has a large effect size. In this region, GABA + concentrations did not differ between groups (*p* = 0.93, Fig. 5g), nor did the Glx/GABA + ratio (*p* = 0.12).

Finally, the GSH concentrations did not differ between groups in the OCC VOI (*p* = 0.98, Fig. 5d) nor in the IFG VOI (*p* = 0.77, Fig. 5h). As GSH plays a role in homeostasis and that an excess of Glx or a deficit in GABA + can lead to excitotoxicity, we assessed whether GSH concentrations were correlated with the other metabolite concentrations. The concentrations of GSH and GABA + in the IFG VOI were negatively correlated (entire sample: *r* = − 0.34, *p* = 0.031). Within group, this correlation was significant in ASD (*r* = − 0.55, *p* = 0.007), but not in NT (*r* = − 0.04, *p* = 0.88). GSH was not correlated with Glx nor GABA + in the OCC VOI, nor with Glx in the IFG VOI (entire sample: p-values > 0.31). These four correlations (on the entire sample) with GSH are not significant when applying FDR corrections for multiple comparisons.

## Discussion

Using an associative learning task and MRS, we examined prediction learning and its molecular correlates in autistic adults. We found that both NT and ASD participants were biased by their expectations, as they tended to report perceiving a rotation consistent with the current contingency in ambiguous trials. Moreover, both groups managed to successfully predict the outcome given the cue and to update their expectations after a change in contingency. This ability to make correct predictions was correlated with the Glx/GABA + ratio in the occipital cortex (positive correlation) and in the right IFG (negative correlation). We also found that the concentration of Glx in the right IFG was increased in ASD individuals compared to NT, whereas the other metabolite concentrations did not differ between groups. As higher Glx/GABA + ratios in the right IFG were associated with lower percentages of correct predictions, the increase in Glx observed in the ASD group might contribute to altered processes of prediction learning in certain contexts.

### Intact prediction learning in autistic adults

Bayesian hypotheses suggest that autistic individuals have difficulties predicting their environment, because priors would be uninformative (10) or because prediction errors would be given a high and inflexible weight [12]. Yet, we found that both NT and ASD adults managed to learn the cue-outcome association and therefore, to deal with some form of expected uncertainty. ASD is also thought to be associated with impairments in context-sensitive adjustments of sensory precision, leading to atypical representations of the environmental volatility [15]. However, in the current experiment, both groups were able to update their beliefs when the association suddenly reversed, suggesting a capacity to deal, to a certain extent, with unexpected uncertainty. Yet, it should be highlighted that participants were explicitly told that the association could change, which therefore reduced the degree of unexpected uncertainty. Given the design of the task (i.e. associations reversing every 16 to 32 trials), participants may have captured the overall underlying structure of the tone-rotation association. In contrast, using the same paradigm [26] but with a lower probabilistic association (62.5% instead of 75%), we had found that autistic adults could learn predictions but did not update their priors after a change in contingency. This difference in results suggests that autistic individuals are able to make predictions in a volatile and uncertain context, but that the amount of uncertainty that can be tolerated to ensure optimal predictive processes is lower in ASD than NT. Notably, a probabilistic reinforcement learning task also indicated an impairment of the ASD group when the probability was set at 70%, but no difference with NT when it was set at 80% [88]. In line with our results, autistic children could update their predictions in a decision-making task in a volatile context [23]. Nonetheless, another associative learning task showed that autistic adults had more difficulties tracking the volatility (which was overestimated), leading to a decreased distinction between expected and unexpected outcomes [24]. In their study, it should be noted that prediction learning was only assessed implicitly, as they did not ask for any prediction response but observed how response times and error rates were modulated by the expectedness of the outcome [24]. Bayesian hypotheses of ASD suggest that the atypical predictive abilities observed in perceptual tasks could be extrapolated to the social domain, which is inherently dynamic and complex. Yet, we did not find significant correlations between predictive abilities and social difficulties in the present study.

In another learning task in a volatile environment, worse performance was associated with more autistic traits in NT [28]. In the present study, the correlation between accuracy levels and autistic traits was non-significant but also negative (small to medium effect size). Yet, contrary to our expectations, individuals with a high intolerance of uncertainty in their daily lives did not have more difficulties to make predictions. It suggests that knowing what to expect is not sufficient to better cope with uncertainty in ASD. Nonetheless, measurements of predictive abilities performed in laboratory conditions are obviously different from real-life situations that are much more complex, noisy and uncertain.

In addition to the explicit prediction response directly assessing whether participants had learned the association, there was an implicit perception response in ambiguous trials that assessed the perceptual bias induced by this expectation. Both NT and ASD individuals were biased by their expectations, as they reported perceiving the rotation that they expected to see, even though there was no rotation. This result does not support the hypo-prior hypothesis stating that priors do not influence perception in ASD [10]. Yet, it should be noted that there was a non-significant trend towards a decreased prior bias in ASD (*d* = 0.47, medium effect), in line with the results of our previous associative learning task in a more uncertain context [26]. We also found that the extent of this prior bias was negatively correlated with the GSQ scores, so that individuals with a more atypical sensory sensitivity (as encountered in ASD) had their percepts less influenced by expectations.

Altogether, these results seem to indicate that the Bayesian hypotheses of ASD need to be refined as autistic adults are able to learn and update their priors in some specific experimental contexts.

### Prediction accuracy is associated with the Glx/GABA + ratios

Based on the predictive coding literature, we had hypothesized that the ability to make correct predictions might be related to the glutamate/GABA balance. The percentages of correct predictions were indeed correlated with the Glx/GABA + ratios in our low-level and higher-level regions of interest: positively correlated in the occipital cortex (medium effect size) and negatively correlated in the right IFG (close to a large effect size).

In other words, individuals who were good at predicting the outcome had high Glx/GABA + ratio in the occipital cortex. This correlation was supported both by an increase in Glx and a decrease in GABA + leading to better performance, and was relatively similar in each group. It suggests that enough excitatory signalling combined with a relatively low inhibition level in the visual cortex ensures typical predictive processing in both groups.

In the right IFG, hypothesized to play a key role in attributing prior and sensory precisions, the Glx/GABA + ratio was higher in individuals who had more difficulties to make correct predictions. This correlation had a large effect size in the ASD group, but a very small effect size in the NT group, and was mostly due to higher Glx concentrations being associated with lower performance. Interestingly, we also found increased Glx levels in this region in ASD, which might imply that an excess of Glx could have prevented from making accurate predictions. As described above, even though the ASD group performed as accurately as the NT group, another experiment with a more uncertain context revealed increased difficulties for ASD adults to update their predictions [24]. We can hypothesize that hyperglutamatergia in the right IFG disturbs the typical predictive mechanisms, by attributing a suboptimal precision, and might contribute to difficulties to flexibly update priors. In line with the hypotheses cited above, it might be interpreted as a disturbed functioning of the predictive brain for high-level aspects (i.e. update of priors across time) in this higher-level region.

### Increased Glx in the right IFG in autistic adults

In the right IFG, Glx levels were higher in ASD than NT, while GABA + concentrations did not differ between groups. To the best of our knowledge, this study is the first one to quantify these metabolite concentrations in the right IFG of autistic adults. A few studies measured them in other frontal regions in the left hemisphere in autistic adults, and showed no difference in GABA + (left ventral premotor cortex, [56]) nor Glx (left dorsolateral prefrontal cortex, [46, 50]). In autistic children, glutamate was found to be increased in a frontal region (unspecified MRS voxel localization) and this measurement was strongly positively correlated with blood glutamate levels [89]. In line with this result, our findings indicate a tendency towards hyperglutamatergia in the right IFG of autistic adults. An excess in glutamate might lead to excitotoxicity and could contribute to imbalanced excitatory transmission in ASD. Following the predictive coding hypotheses, it would alter the encoding of prediction errors and therefore the update of priors. Even though Glx measurements performed with MRS are noisy and composite (glutamate and glutamine), an increase in Glx might also lead to an increased excitatory/inhibitory balance. This balance is crucial for optimal neural signal formation and transmission, and several theories suggest that an increased in this balance might underlie the abnormalities encountered in ASD [90–92]. In the occipital cortex, the absence of group difference in GABA + or Glx concentrations is in line with the literature (GABA + : [49, 52, 55, 56]; Glx: [49, 52]).

In addition to this potentially disturbed excitatory/inhibitory balance, an imbalance between oxidative stress and antioxidant systems can destabilize neural networks. GSH is the main free radical scavenger in the brain and therefore protects neurons. Using the HERMES sequence, GSH was simultaneously edited with Glx and GABA + . GSH concentrations did not differ between groups in the occipital cortex or in the IFG, suggesting typical antioxidant functions in ASD. In the right IFG only, high GSH concentrations were associated with low GABA + concentrations in autistic adults. This correlation might underlie the fact that both GABA and GSH have glutamate as precursor, and that if glutamate is mostly transformed into GSH, then it will be associated with less GABA synthesis. It might also suggest that in the right IFG of autistic adults, synthesizing glutathione to protect neurons may be done at the expense of GABA inhibition.

As higher glutamate/GABA ratios in the IFG were associated with worse predictive abilities and that autistic individuals had more glutamate in this region, we can hypothesize the encoding of priors and prediction errors might be altered in the IFG in ASD. It might, in turn, lead to difficulties to learn certain statistical regularities of the environment (such as, its volatility) in ASD when the uncertainty is high (e.g. [26]) but not when it is less uncertain (current study). Yet, we highlight that this interpretation is currently only a hypothesis, that other neurotransmitters [4, 13, 34, 93] might come at play in encoding predictions and that we only measured concentrations but did not assess any other aspects of the glutamatergic or GABAergic pathways. Furthermore, the hypotheses regarding the role of glutamatergic/GABAergic balance in predictive coding are specific to different layers of the cortex, while MRS only allowed measurements in a large brain region (obviously including all cortical layers).

### Limitations

Limitations of these MRS measurements include that GABA might be co-edited with macromolecules [94], that glutamate is co-edited with glutamine, and that to ensure a good fit quality, we had to reduce our sample size for certain measurements. Furthermore, our examination of GSH was exploratory, since HERMES allows for simultaneous measurement of GABA, Glx, and GSH, which contribute to the same metabolic pathway. In addition, there is some evidence of altered GSH metabolism in ASD (e.g. [64]). However, there is limited consensus regarding fitting and quantification of GSH. While peak-based fitting approaches for the GABA difference spectrum are widely used, given the complexity of the GSH difference spectrum, peak-based fitting approaches are perhaps less optimal (see also: [85]). Similarly, while for quantification of GABA, there is substantial evidence for tissue-specific relaxation and concentration differences, this is less obvious for GSH. In Gannet 3.1 the approach for GSH is identical to that of GABA. In the future, better fitting approaches and quantification approaches for GSH are required, for example, linear combination model fitting [95]. Furthermore, in the future, we will acquire both short TE and metabolite-TE water unsuppressed data for both quantification and eddy current correction as is now recommended [84, 96]. In order to minimize the scanning time, we focused on two regions only, but measurements in other regions such as the left orbitofrontal cortex, the anterior cingulate cortex or the cerebellum could have been informative given their role in signalling predictions or prediction errors. Moreover, eleven participants with ASD were taking one or several medications, which might have affected some of the results reported in this manuscript. Future studies should replicate these findings using larger samples to assess whether the absence of significant differences is due or not to the small sample size.

## Conclusions

Neurotypical and autistic adults successfully learned and updated predictions, suggesting no particular impairment of the predictive brain in ASD in this context. The Bayesian hypotheses of ASD should be refined, as uniformly weak priors do not seem to accurately describe their behaviour. MRS results indicated that an excess in glutamate in the right IFG in autistic adults could be associated with suboptimal predictive mechanisms. In order to directly assess the role of glutamatergic/GABAergic neurotransmission in learning priors, future studies could use glutamatergic antagonists or transient disruption of GABAergic signalling to evaluate their effect on prediction learning in ASD.

## Supplementary Information

**Additional file 1**. Post-experiment debriefing.

## Data Availability

The datasets used and/or analysed during the current study are available from the corresponding author on reasonable request.

## Abbreviations

AQ: Autism-spectrum Quotient
ASD: Autism Spectrum Disorder
CCW: Counterclockwise
CW: Clockwise
GABA: Gamma-aminobutyric acid
Glx: Glutamate and glutamine
GSH: Glutathione
HERMES: Hadamard Encoding and Reconstruction of MEGA-Edited Spectroscopy
HIPPEA: High and Inflexible Precision of the Prediction Error in Autism
IFG: Inferior frontal gyrus
MRS: Magnetic resonance spectroscopy
NMDA: N-methyl-D-aspartate
NT: Neurotypical
OCC: Occipital cortex
TE: Echo time
TR: Repetition time
VOI: Volume of interest
