# Supplementary material for: Prediction learning in adults with autism and its molecular correlates
Source: Mol Autism. 2021 Oct 6;12:64. doi: 10.1186/s13229-021-00470-6 (PMC8493731; doi:10.1186/s13229-021-00470-6)
Supplement: Supplementary file 1 — Additional file 1. Post-experiment debriefing. [file 13229_2021_470_MOESM1_ESM.docx]

# Supplementary information

### SI.1. Post-experiment debriefing

On a 0 to 10-point scale (i.e., very easy to very difficult), the task difficulty score was rated as 5.1 (± 2.3) in the NT group and 5.2 (± 2.7) in the ASD group (no group difference, *p* = .93). The proportion of participants reporting that they noticed that the dots sometimes did not turn was 42% in the NT group and 58% in the ASD group (no proportion difference, *p* = .75). We compared two subgroups: those reporting that they noticed that the dots sometimes did not rotate with those who did not notice it. The percentages of ambiguous trials perceived as rotating according to the contingency did not differ between these two subgroups (69% ± 12 vs. 70% ± 13, *p* = .74).
